# New insights into *Ituglanis* (Siluriformes: Trichomycteridae) diversity in Bahia State, eastern Brazil: Description of a new species and conservation status reappraisal

**DOI:** 10.1111/jfb.70246

**Published:** 2025-10-05

**Authors:** Paulo J. Vilardo, Axel M. Katz, Wilson J. E. M. Costa

**Affiliations:** ^1^ Laboratory of Systematics and Evolution of Teleost Fishes, Institute of Biology, Zoology Department Federal University of Rio de Janeiro, Centro de Ciências da Saúde – Cidade Universitária Rio de Janeiro Brazil

**Keywords:** Atlantic Forest, biodiversity, catfish, conservation status, Neotropics, systematics

## Abstract

The northeastern Atlantic Forest of Brazil harbours a remarkable diversity of species of Trichomycteridae, many of which remain poorly understood. This study describes a new species of *Ituglanis*, endemic to the Ribeirão São Roque, a stream tributary of Rio Piabanha, an affluent of Rio Colônia basin, Bahia State, Brazil. The new species is distinguished from its congeners by a combination of the following characters: four pairs of ribs; 35 post‐Weberian vertebrae, seven pectoral‐fin rays; and a colour pattern consisting of irregular dark brown spots along the flanks and dorsal region. The specimens were encountered during field studies conducted in southern Bahia State aimed at collecting *Ituglanis cahyensis* and *Ituglanis agreste.* However, despite extensive efforts, neither of these species was found during surveys, suggesting potential population declines. Additionally, we reassess the conservation status of the species of *Ituglanis* from Bahia State, proposing status updates for three of the four previously described species based on recent field data and habitat assessments. The discovery of this new species underscores the urgent need for further exploration and collection in poorly sampled areas of the Atlantic Forest. Human activities, including deforestation and habitat degradation, continue to threaten these unique ecosystems. As demonstrated here, the description of new species contributes not only to a better understanding of biodiversity and evolutionary relationships within *Ituglanis* but also to conservation efforts in one of the most endangered biodiversity hotspots in the world.

## 1 INTRODUCTION

The genus *Ituglanis* Costa & Bockmann, 1993 represents a monophyletic group of small trichomycterids distributed across almost all major South American drainages (Costa et al., 2021). Established to include species formerly assigned to *Trichomycterus* Valenciennes, 1832, *Ituglanis* has emerged as one of the most species‐rich genera within Trichomycteridae (Costa et al., 2021). Over the past two decades, the number of valid species in the genus has nearly doubled, increasing from 15 to 31 (Fricke et al., 2025). This rapid growth in species descriptions reflects not only the remarkable diversity of the genus but also the ongoing efforts to resolve its complex taxonomy.

Despite significant progress in understanding *Ituglanis*, the genus diversity and evolutionary relationships remain poorly characterized. Although many described species exhibit endemic distributions restricted to a single river basin [e.g., *Ituglanis apteryx* Datovo, 2014, *Ituglanis crispim* Donin, de Pinna, Severi & Ramos, 2023, *Ituglanis inusitatus* Ferrer & Donin, 2017 and *Ituglanis parkoi* (Miranda‐Ribeiro, 1944)], others such as *Ituglanis amazonicus* (Steindachner, 1882), *Ituglanis eichorniarium* (Miranda‐Ribeiro, 1912) and *Ituglanis goya* Datovo et al., 2016 show broader geographical ranges. The genus also displays remarkable ecological diversity, with some species inhabiting subterranean environments and showing unique troglobitic adaptations (Rizzato & Bichuette, 2014). At least one species, like *Ituglanis compactus* Castro & Wosiacki, 2017, represents a possible case of miniaturization. Despite this remarkable diversity in both ecological adaptations and morphological forms, the diagnosis of *Ituglanis* comprises a unique combination of morphological characters applicable to all species: (1) a reduced or absent posterior cranial fontanel; (2) an anteriorly directed anterolateral extremity of the sphenotic‐prootic‐pterosphenoid complex; (3) a long lateral process of the parurohyal; (4) a deep medial concavity on the autopalatine; (5) an approximately semi‐circular metapterygoid, with an antero‐dorsal convex margin; and (6) two to eight ribs (Costa et al., 2021). However, overlaps in morphological characters with related genera, such as *Trichomycterus*, make complex the visual species identification in the field. For instance, *Ituglanis payaya* (Sarmento‐Soares et al., 2011) was originally described as a species of *Trichomycterus*, but a recent integrative analysis combining morphology and molecular phylogeny repositioned it within *Ituglanis*. Costa et al. (2021) demonstrated that this species, from Chapada Diamantina, in Bahia State, is closely related to *Ituglanis paraguassuensis* Campos‐Paiva & Costa, 2007 from the same region. These findings underscore the importance of combining morphological and molecular data to resolve taxonomic ambiguities and better understand the evolutionary relationships within the genus.

The species described here was found during a field expedition conducted in March 2024 in the remnants of the Atlantic Forest of southern state of Bahia, northeastern Brazil. The primary objective of this expedition was to collect material for molecular analyses of other species from southern Bahia State, *Ituglanis cahyensis* Sarmento‐Soares et al., 2006 and *Ituglanis agreste* Lima et al., 2013, both described from this same phytogeographical area (Camelier & Zanata, 2014). However, during this survey, specimens of an undescribed species were collected, prompting the present study. Notably, this population had already been reported in a fish inventory of southern Bahia (Cetra et al., 2010). At the time of collection, we hypothesized that it might correspond to one of the species already described for the region. Nonetheless, further investigation confirmed it to be a distinct, undescribed species.

The Atlantic Forest is one of the most diverse and threatened biodiversity hotspots in the world, originally covering about 150 million hectares through almost all the coast of Brazil and portions of Argentina and Paraguay (Ribeiro et al., 2009; Vancine et al., 2024). The remnants of the Atlantic Forest play a crucial role in fish conservation, hosting over 270 freshwater fish species distributed among more than 90 genera and 20 families (Abilhoa et al., 2011; Miranda, 2012). Although this ecosystem shelters a rich biodiversity, decades of deforestation and anthropogenic actions have reduced its native vegetation to less than 13% of its original coverage (Morellato & Haddad, 2000). Some of the key Atlantic coastal drainages include, from north to south, the Rio Paraguaçu, Rio de Contas, Rio Pardo and Rio Doce basins, which are characterized by complex headwater systems that are separated from the Rio São Francisco basin by the highlands of the Espinhaço complex (Camelier & Zanata, 2014).

Herein, we provide a formal description of the new taxon found in remnants of the Atlantic Forest and re‐evaluate the conservation status of *Ituglanis* species from Bahia State.

## 2 MATERIALS AND METHODS

Measurements and counts follow Costa (1992), with modifications proposed by Costa et al. (2020). Measurements are presented as percentages of standard length (SL) except for subunits of head, which are presented as percentages of head length (HL). All measurements and counts were made on the left side of specimens whenever possible. Counts of procurrent caudal‐fin rays, vertebrae, branchiostegal rays and odontodes were made only in cleared and stained (C&S) specimens prepared according to Taylor and Van Dyke (1985). Anatomical illustrations were prepared based on sketches made using a stereomicroscope with camera lucida. Terminology for osteological nomenclature followed Bockmann et al. (2004) and modifications proposed by Costa (2021). Terminology for the cephalic laterosensory system followed Rizzato and Bichuette (2016), but nomenclature for pores also follows Arratia and Huaquin (1995). Morphological and cephalic laterosensory system information was obtained through personal observations of the analysed species, original descriptions and observations documented by Rizzato and Bichuette (2016). A list of comparative material is provided in Costa et al. (2021); an additional list of material is described in Supporting Information S1.

The conservation status of species of *Ituglanis* from Bahia State was assessed based on the International Union for Conservation of Nature (IUCN) criteria (IUCN Standards and Petitions Subcommittee, 2022) following ‘Freshwater Species Mapping Standards for IUCN Red List Assessments’, the Annex I of the ‘Mapping Standard and Data Quality for IUCN Red List Spatial Data’. The extent of occurrence (EOO) was determined using the minimum convex polygon drawn around each location where each species is known to occur. The total area of each microbasin was calculated using QGIS LTR 3.40 with the HydroBASINS level 12 dataset (Lehner & Grill, 2013).

### 2.1 Ethics statement

Specimens were captured using small dip‐nets (40 × 30 cm) during daylight. Collections were made using permits provided by ICMBio (Instituto Chico Mendes de Conservação da Biodiversidade; permit number: 38553‐13). Specimens were killed just after collection by submerging them in a buffered solution of ethyl‐3‐amino‐benzoat‐methansulfonat (MS‐222) at a concentration of 250 mg/L, for a period of 10 min or more, until completely ceasing opercular movements, following the methods for euthanasia approved by CEUA‐CCS‐UFRJ (Ethics Committee for Animal Use of Federal University of Rio de Janeiro; permit number: 084/23). Specimens used for morphological comparisons were fixed in buffered formalin for a period of 14 days and then transferred to 70% ethanol. In lists of material, the abbreviations C&S indicate specimens prepared for osteological analysis and preserved in glycerine (see below), and DNA indicates specimens fixed and preserved in 98% ethanol. Specimens were deposited in the fish collection of the Institute of Biology, Federal University of Rio de Janeiro (UFRJ).

## 3 RESULTS

### 3.1 Taxonomic accounts

*Ituglanis jussariensis* sp. n. Vilardo, Katz and Costa.

urn:lsid:zoobank.org:act:7AD5F948‐28C2‐41D8‐9CDD‐C9F71D667ACE.

*Ituglanis* sp.: Cetra et al., 2010.

**FIGURE 1:**
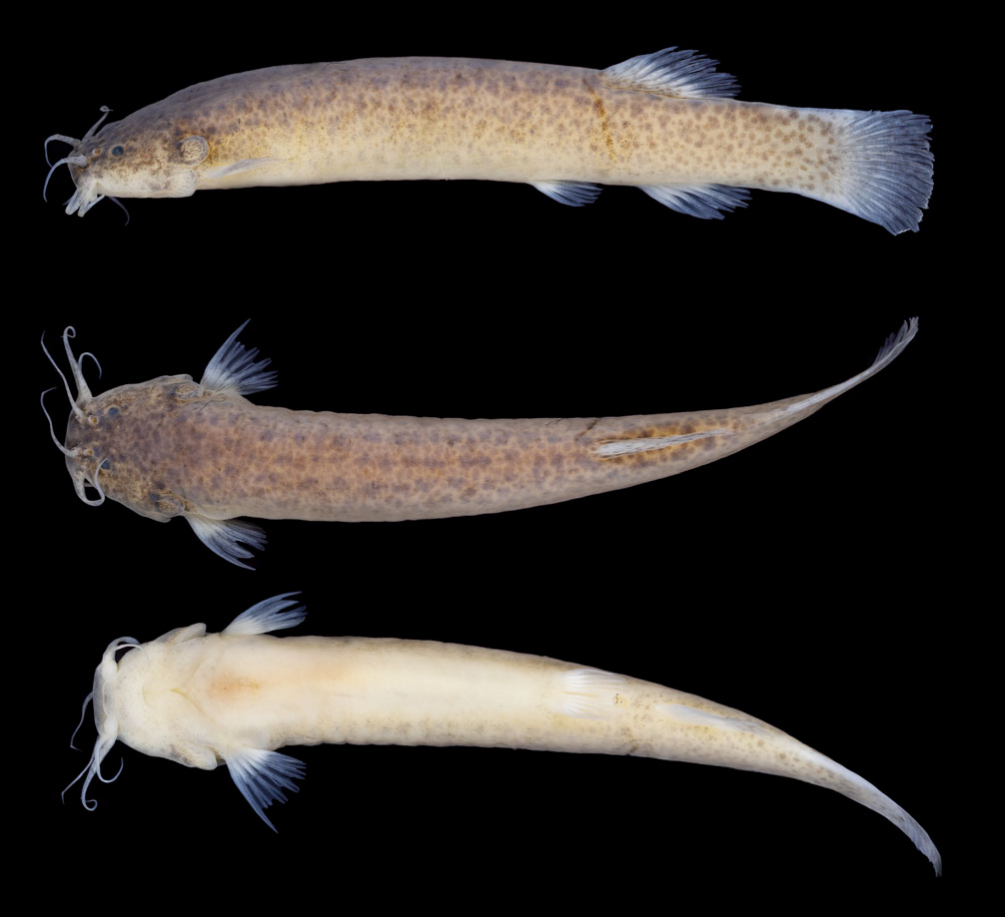
*Ituglanis jussariensis* holotype. UFRJ 14477, 42.0 mm SL, stream tributary to Rio São Roque drainage, Rio Piabanha basin. Left lateral, dorsal and ventral views.

**FIGURE 2:**
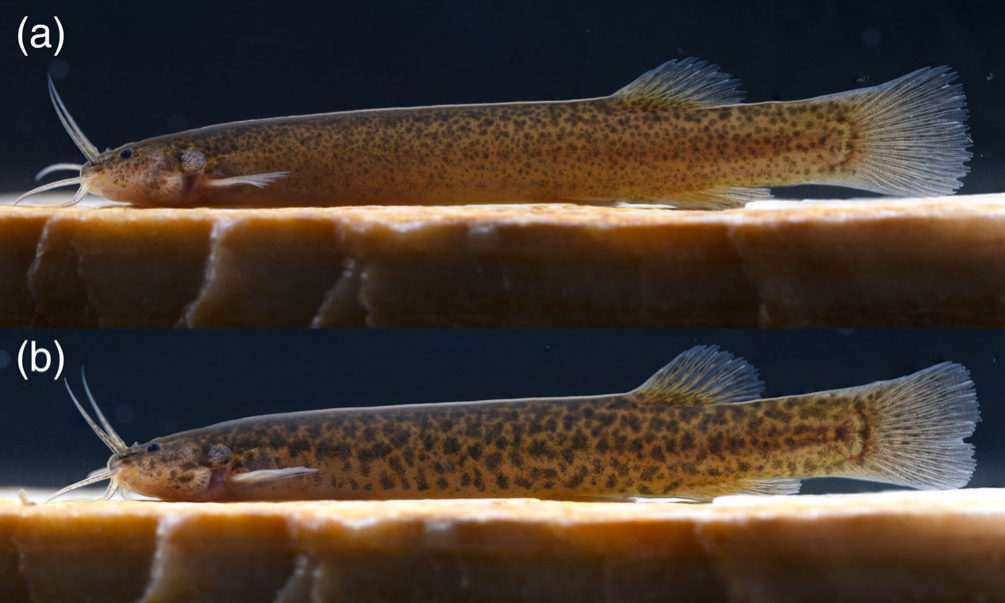
*Ituglanis jussariensis* live holotype and paratype. (a) UFRJ 14477, holotype, 42.0 mm SL. (b) UFRJ 13135, paratype, 44.0 mm SL.

**FIGURE 3:**
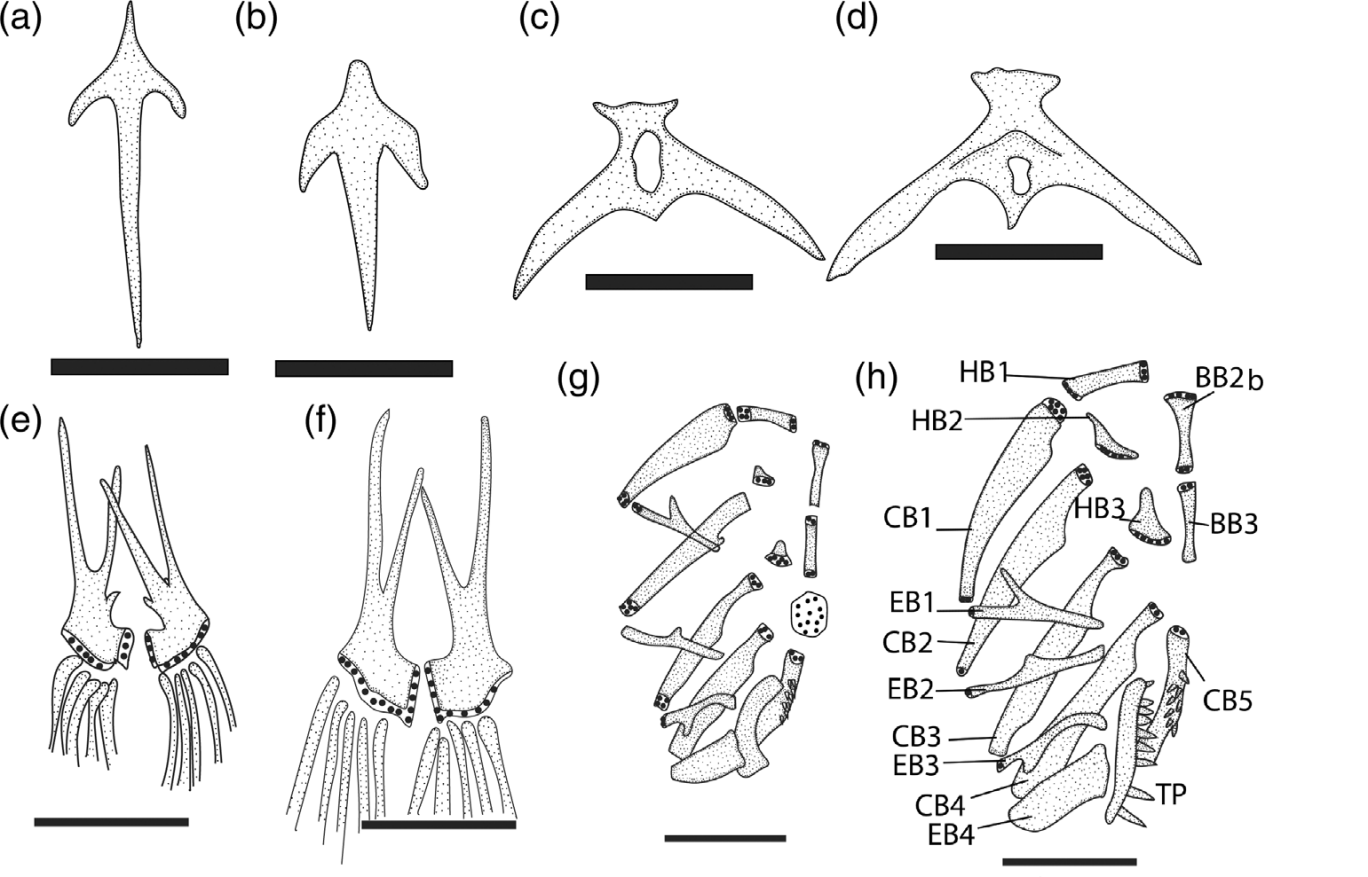
Osteological features in *Ituglanis* species. (a,b) Vomer, ventral view: (a) *I. jussariensis* and (b) *Ituglanis paraguassuensis*. (c,d) Parurohyal, dorsal view: (c) *I. paraguassuensis* and (d) *Ituglanis jussariensis*. (e,f) Pelvic bone, ventral view: (e) *I. paraguassuensis* and (f) *I. jussariensis*. (g,h) Branquial skeleton, dorsal view: (g) *I. paraguassuensis* and (h) *I. jussariensis*. BB2–4, basibranchials 2 to 4; CB1–5, ceratobranchials 1 to 5; EB1–4, epibranchials 1 to 4; HB1–3, hypobranchials 1 to 3; TP, tooth plate. Larger stippling represents cartilages.

**FIGURE 4:**
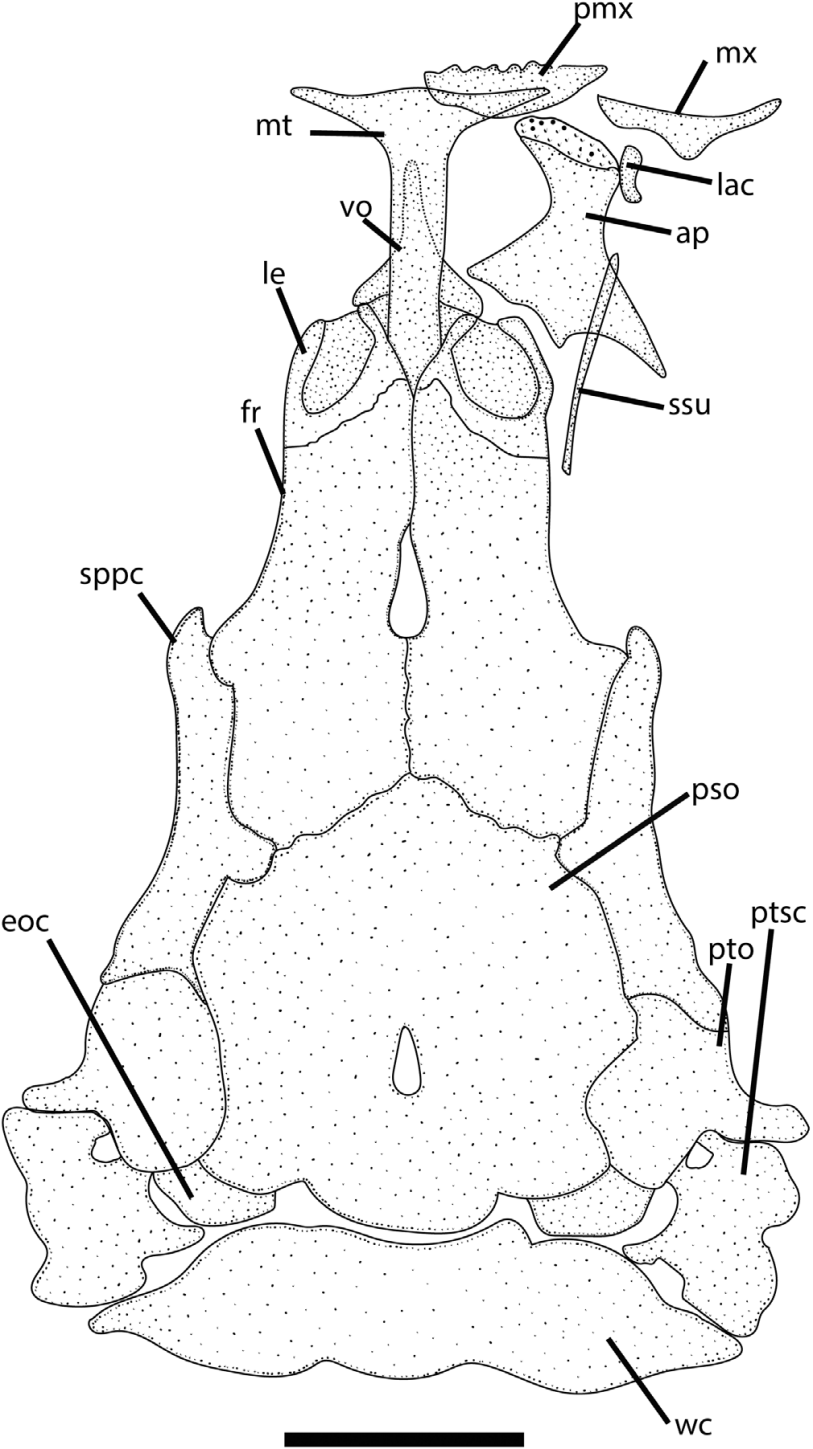
Dorsal view of neurocranium of *Ituglanis jussariensis*. UFRJ 14160, 37.1. mm SL. Ap, autopalatine; eoc, epioccipital; fr, frontal; lac, lacrimal; Mt., mesethmoid; Mx, maxila; Pmx, premaxilla; Pso, parieto‐supraoccipital; Ptsc, posttemporosupracheithrum; Sppc, sphenotic‐prootic‐pterosphenoid complex; Ssu, sesamoid supraorbital.

**FIGURE 5:**
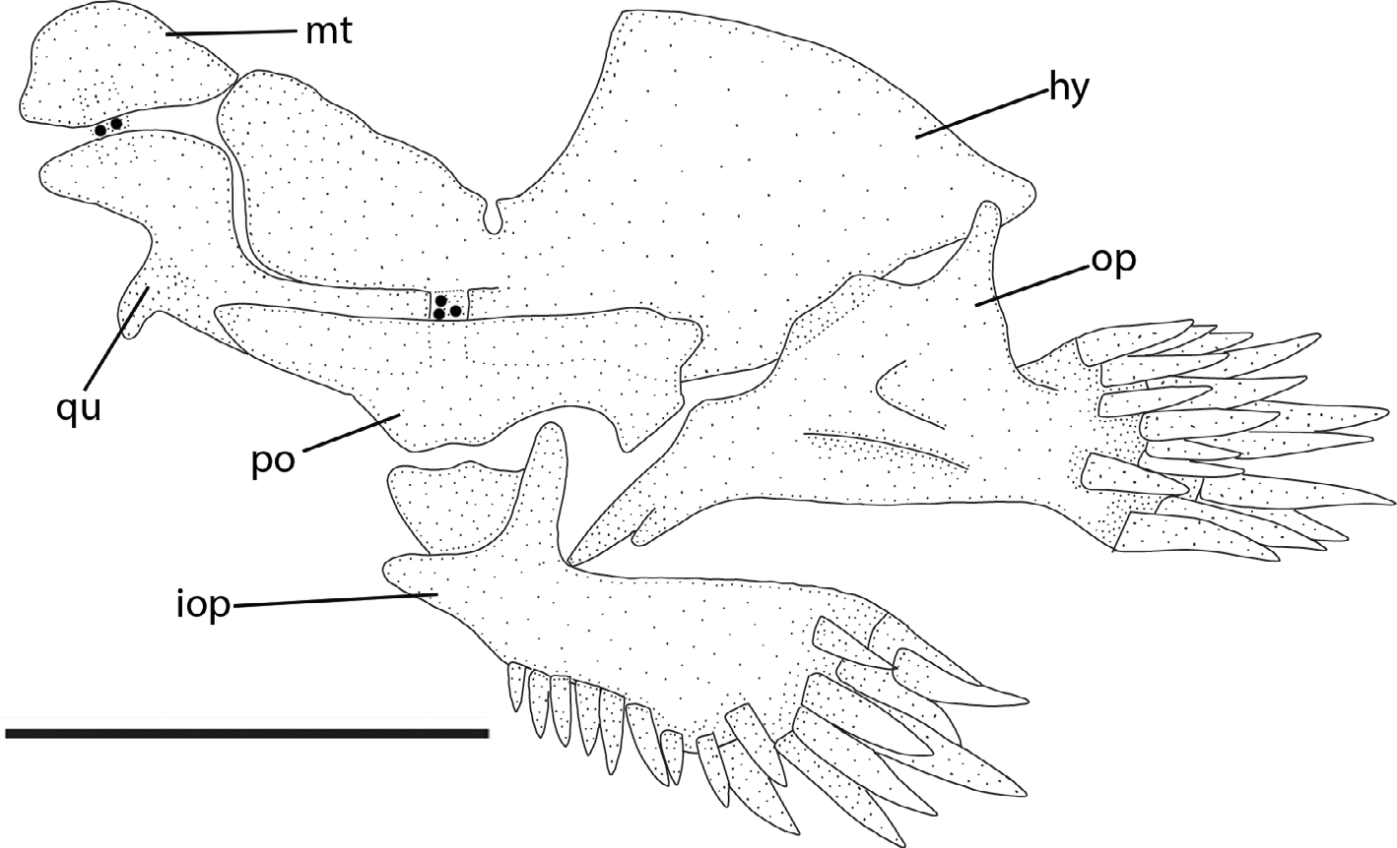
Left suspensorium and opercular series of *Ituglanis jussarienis*. UFRJ 14160, 37.1 mm SL. Lateral view. Hy, hyomandibula; iop, interopercle; mt, metapterygoid; op, opercle; po, preopercle; qu, quadrate. Larger stippling represents cartilages.

**FIGURE 6:**
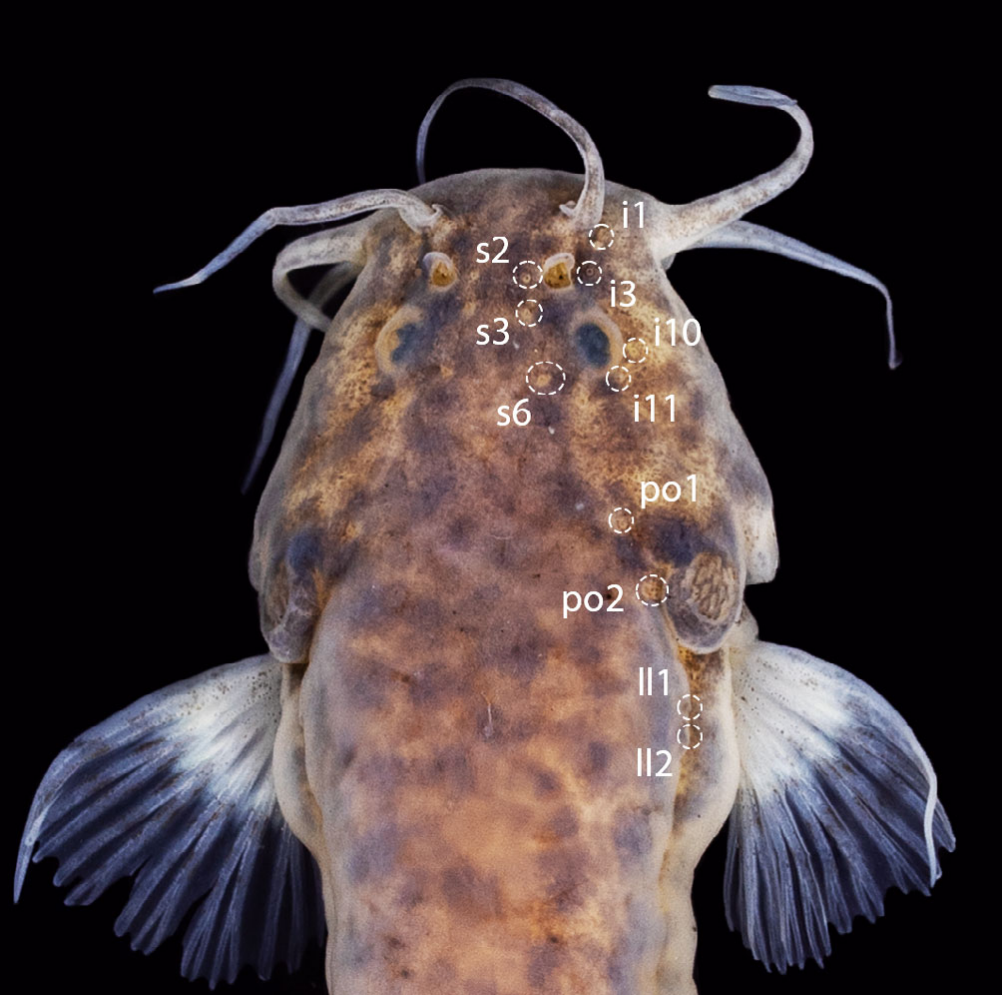
Dorsal view of head of *Ituglanis jussariensis*. UFRJ 13135, paratype, 44.0 mm SL. Latero‐sensory pores are indicated by white circles.

Figures 1, 2, 3, 4, 5, 6; Table 1.

**TABLE 1:** Morphometric data of *Ituglanis jussariensis* sp. nov.

	Holotype	Paratypes (*n* = 5)	Mean	Standard deviation
Standard length (mm)	42.0	31.0–44.6	37.46	5.3
Percentage of standard length				
Body depth	13.8	13.1–14.4	13.8	0.5
Caudal peduncle depth	12.1	11.6–12.3	11.9	0.3
Body width	7.4	7.2–8.53	7.7	0.6
Caudal peduncle width	1.6	1.3–2.1	1.6	0.3
Predorsal length	68.4	66.8–72.3	69.7	1.9
Prepelvic length	57.8	57.7–61.35	59.5	1.3
Dorsal‐fin base length	11.2	8.4–11.1	10.0	1.0
Anal‐fin base length	8.4	6.6–9.8	8.2	1.1
Caudal‐fin length	15.7	15.4–17.4	16.5	0.9
Pectoral‐fin length	10.7	9.9–11.3	10.6	0.5
Pelvic‐fin length	9.6	8.4–9.6	9.1	0.4
Head length	16.7	16.6–19.4	18.4	1.1
Percentage of head length				
Head depth	62.8	48.5–63.2	55.5	6.5
Head width	98.5	81.9–98.5	87.6	6.1
Snout length	37.1	32.0–37.8	34.7	2.4
Interorbital length	28.5	24.7–41.9	26.8	2.1
Preorbital length	18.3	15.8–19.3	17.7	1.4
Eye diameter	16.4	12.2–16.4	14.8	1.6

### 3.2 Holotype

UFRJ 14447, 42.0 mm SL; Brazil: Bahia State: Jussari Municipality: Ribeirão São Roque, a stream tributary of Rio Piabanha, an affluent of Rio Colônia basin, 15°12′ 53.02″ S 39°31′13.63″ W, about 1130 m asl; A. Katz and P. Vilardo, 26 March 2024.

### 3.3 Paratypes

UFRJ 14135, 3 ex., 44.0–31.0 mm SL; UFRJ 14160, 2 ex. (C&S), 32.0–37.1 mm SL mm SL; UFRJ 14089, 2 ex. (DNA), 27.4–29.0 mm SL; all collected with the holotype.

### 3.4 Diagnosis

*I. jussariensis* is distinguished from all congeners by the combination of the following characters: four pairs of ribs; 35 post‐Weberian vertebrae, seven pectoral‐fin rays; and a colour pattern consisting of irregular dark brown spots along the flanks and dorsal region (Figures 1 and 2). The presence of four pairs of ribs (vs. three or less) distinguishes the new species from all congeners except: *I. agreste*, *Ituglanis amphipoptamus* Mendonça, Oyakawa & Wosiacki, 2018; *Ituglanis australis* Datovo & de Pinna, 2014, *Ituglanis bambui* Bichuette & Trajano, 2004, *Ituglanis boitata* Ferrer, Donin & Malabarba, 2015, *Ituglanis boticario* Rizzato & Bichuette, 2015, *I. cahyensis*, *Ituglanis epikarsticus* Bichuette & Trajano, 2008, *I. goya*, *Ituglanis guayaberensis* (Dahl, 1960), *Ituglanis mambai* Bichuette & Trajano, 2008, *I. paraguassuensis*, *Ituglanis parahybae* (Eigenmann, 1918), *Ituglanis passensis* Fernández & Bichuette, 2002, *Ituglanis proops* (Miranda‐Ribeiro, 1908), *I. payaya* and *Ituglanis ramiroi* Bichuette & Trajano, 2004. The presence of I,6 pectoral‐fin rays distinguishes *I. jussariensis* from *I. cahyensis* and *I. parahybae* (vs. I,4), *I. amphipoptamus* and *I. australis* (vs. I,5), *I. bambui, I. epikarsticus, I. guayaberensis, I. mambai and I. passensis* (vs. I,7), *I. ramiroi* (vs. I,8) and *I. boticario* (I,7–8). *I. jussariensis* is distinguished from *I. boitata* by the fewer number of vertebrae (35 vs. 41–42) and ribs (4 vs. 5–6). *I. jussariensis* is further distinguished from *I. proops* by the size and position of the interopecular patch of odontodes in relation to the opercular patch of odontodes (similar in size and distal end of interopercular patch of odontodes not reaching a transverse line through the anterior margin of opercular patch of odontodes vs. almost double the size, passing to the middle of the opercular patch of odontodes) (Mendonça et al., 2018: Figure 3b). *I. jussariensis* is distinguished by *I. goya* by having a fewer number of post‐Weberian vertebrae (35 vs. 39–42) and by colour pattern (irregular dark brown spots along flanks and dorsal region vs. dark longitudinal stripes along the trunk and many spots scattered on the flanks and dorsal region). *I. jussariensis* is distinguished from *I. agreste*, its geographically closest species, by a fewer number of post‐Weberian vertebra (35 vs. 36), a fewer number of interopercular odontodes (23–24 vs. 26–30), by a fewer number of ribs (4 vs. 5–6), by the dorsal‐fin origin at the vertical through the 19th vertebra (vs. 22nd vertebra), by a large parurohyal foramen longitudinally elongated and positioned along almost all the longitudinal parurohyal length (Figure 3c vs. a thick parurohyal foramen positioned at the posterior portion of parurohyal, Lima et al., 2013: Figure 5), by cranial fontanels drop like with the anterior fontanel almost two times bigger than the posterior fontanel (Figure 4, vs. anterior fontanel restricted to a small pit and posterior fontanel as small round opening, Lima et al., 2013: Figure 2a), by maxilla and premaxilla bones with almost the same length (Figure 4, vs. maxilla shorter than premaxilla, Lima et al., 2013: Figure 2a) and by the smaller and equilateral triangular shape of the hypobranchial 2–3 (Figure 3g, vs. elongated and isosceles triangular shape of the hypobranchial 2–3, Lima et al., 2013: Figure 6). *I. jussariensis* is distinguished from *I. paraguassuensis* by the morphology of its lateral ethmoid with lateral margin forming a distinct wall limiting the fossa aperture (Figure 4, vs. absence of a distinct wall limiting the fossa aperture, Campos‐Paiva & Costa, 2007: Figure 2), by a smaller pentagonal epioccipital (Figure 4, vs. bigger quadrangular epioccipital with a lateral process, Campos‐Paiva & Costa, 2007: Figure 2), by a slender and pointed vomer (Figure 2a, vs. a broad vomer with rounded extremities, Figure 3b), by a large parurohyal foramen longitudinally elongated and positioned along almost all the longitudinal parurohyal length (Figure 3c, vs. rounded parurohyal foramen positioned in the posterior portion of parurohyal, Figure 3d), by the smaller and equilateral triangular shape of the hypobranchial 2–3 (Figure 3g, vs. elongated and isosceles triangular shape of the hypobranchial 2–3, Figure 3h), by the presence of an anterior medium process in the pelvic girdle (Figure 3e, vs. absence, Figure 3f) and by the presence of irregularly arranged blotches on the flank (vs. irregular pale brown blotches longitudinally aligned along the body). *I. jussariensis* is further distinguished from *I. payaya* by having fewer ribs (4 vs. 5–6) and by its reduced and drop‐like posterior cranial fontanel (Figure 4, vs. a broad posterior cranial fontanel, Sarmento‐Soares et al., 2011: Figure 2).

### 3.5 Description

#### 3.5.1 External morphology (Figures 1 and 2)

Morphometric data are presented in Table 1. Body moderately slender and subcylindrical (Figure 2). Greatest body depth at vertical immediately in front of dorsal‐fin base. Dorsal profile ranging from straight to slightly convex between snout and dorsal‐fin origin, straight on caudal peduncle; ventral profile straight to slightly convex between lower jaw and anal‐fin origin, slightly concave on caudal peduncle. Dorsal and anal fins subtriangular, distal border slightly convex. Dorsal‐fin origin located slightly anterior to midbody. Pectoral fin subtriangular in dorsal view, first pectoral‐fin ray terminating in a short filament reaching about 10%–30% of pectoral‐fin length without filament. Pelvic fin sub‐truncate, with slightly convex posterior margin; its posterior extremity just surpassing urogenital papilla. Pelvic‐fin bases medially separated by minute interspace. Caudal fin rounded. Anus and urogenital papilla at vertical through dorsal‐fin origin, nearer tip of pelvic fin than anal‐fin origin. Head trapezoidal in dorsal view. Eye moderate, dorsally positioned on head. Posterior nostril located closer to anterior orbital rim than anterior nostril. Tip of maxillary barbel reaching area between opercle and preopercle. Tip of rictal barbel reaching anterior margin of opercular patch of odontodes. Tip of nasal barbel reaching anterior margin of opercular patch of odontodes. Mouth subterminal. Jaw teeth pointed and arranged in irregular rows, two rows in premaxilla, two in dentary; premaxillary anterior‐most row with 7–9 teeth, dentary external row with 11–13.

#### 3.5.2 Neurocranium (Figures 3a and 4)

Anterior margin of mesethmoid straight to slightly convex, mesethmoid cornua slender, tip rounded to slightly pointed, reaching two‐thirds of premaxilla. Lateral ethmoid without lateral projections, lateral margin forming distinct wall limiting fossa aperture. Frontal and parieto‐supraoccipital fully joined by sutures; fontanels drop‐like, anterior fontanel two times longer than posterior fontanel. Sphenotic‐prootic‐parasphenoid co‐ossified with anterior projection sheltering infraorbital sensory canal opening. Vomer slender, arrow‐shaped, with long posterior process. Sesamoid supraorbital elongated and narrow, its length about four or five times lacrimal length. Premaxilla subretangular in dorsal view, its lateral extremity slightly pointed. Maxilla boomerang‐shaped, approximately same size as premaxilla. Autopalatine with medial margin deep concave and lateral margin concave. posterolateral process of autopalatine triangular, its length about two‐thirds of autopalatine length without process, extending over metapterygoid.

#### 3.5.3 Jaw suspensorium and opercular series (Figure 5)

Metapterygoid large, about semicircular, with posterior margin weakly pointed towards hyomandibular. Quadrate L‐shaped with large anterior process expanded anteriorly, but not posteriorly, and without contact with the anterior margin of hyomandibula. Hyomandibula well developed with deep notch in dorsal margin. Preopercle long, robust. Opercle robust with small process directed dorsally and 16–18 conical odontodes. Interopercle elongated with 23–24 conical odontodes positioned just under dorsal process and extending through posterior margin.

#### 3.5.4 Hyoid and branchial arches (Figure 3c,g)

Branchiostegal rays 7, or 7 rays plus rudimentary in one specimen. Ventral hypohyal triangular. Anterior ceratohyal elongate, slight expanded in both tips. Posterior ceratohyal triangular and wider than ventral hypohyal. Parurohyal with lateral processes relatively elongate and pointed. Parurohyal head well developed with prominent anterolateral paired processes. Parurohyal foramen large, longitudinally elongated and positioned along almost all the longitudinal parurohyal length. Posterior parurohyal process minute (Figure 3c). Basibranchial 2 and 3 connected by cartilage, similar in width, basibranchial 2 slightly longer. Basibranchial 4 hexagonal and entirely cartilaginous. Hypobranchial 1 elongated with cartilage at both tips. Hypobranchial 2 and 3 small and equilateral triangular shape. Ceratobranchial 1 robust, proximal portion wider than distal one; ceratobranchial 2 slightly widened in its middle portion, with slight concavity on posterior margin of proximal portion; ceratobranchial 3 similar to ceratobranchial 2, except by deep concavity on posterior margin of proximal portion; ceratobranchial 4 subrectangular, slightly widening distally; ceratobranchial 5 with approximately 10 teeth. Epibranchial 1,2 and 3 slender; epibranchial 4 broad, subretangular, proximal portion broader than distal portion. Pharyngobranchial 3 short, subcylindrical; pharyngobranchial 4 long, bearing broad dentigerous plate with curved conical teeth (Figure 3g).

#### 3.5.5 Axial skeleton

Total dorsal‐fin rays 11 or 12 (iii + II + 6–7); dorsal‐fin origin in vertical through centrum of 19th vertebra. Total pectoral‐fin rays 7 (I + 6). Total pelvic‐fin rays 5 (I + 4); pelvic bone with two anterior processes and presence of a small medial process (Figure 3e). Total anal‐fin rays 10 (iii + II + 5); anal‐fin origin in vertical between centrum of 21st or 22nd vertebrae, just posterior to 4th dorsal‐fin ray. Total principal caudal‐fin rays 13 (I + 5 + 6 + I), total dorsal procurrent rays 13–16 (xiii–xvi), total ventral procurrent rays 13 (xiii). Vertebrae 35. Ribs 4.

#### 3.5.6 Laterosensory system (Figure 6)

Laterosensory canals with simple branches ending in single pores. Nasal and frontal canals of supraorbital line continuous, with three pores: s2, adjacent to medial margin of posterior nostril; s3, just posterior to posterior margin of posterior nostril; and s6, in transverse line through posterior portion of orbit. Antorbital segment of infraorbital and sphenotic canals of infraorbital line with two pores each: anterior segment with pore i1, in transverse line through anterior nostril, and pore i3, in transverse line just anterior to posterior nostril; sphenotic canal with pore i10, adjacent to ventral margin of orbit, and pore i11, just posterior to orbit (Figure 6). The otic, postotic, scapular and trunk canals are continuous to each other. Preopercle‐mandibular branch of otic canal ending in po1 pore in vertical line above posterior portion of interopercular patch of odontodes; and the pterotic branch ending in po2 pore in vertical line above posterior portion of opercular patch of odontodes. Trunk canal short with two pores (ll1, ll2) with anterior‐most pore at vertical just posterior to pectoral‐fin base.

#### 3.5.7 Colouration in life (Figure 2)

Flank brownish yellow with diffuse irregularly round‐shaped dark brown dots. Bigger dots more concentrated on dorsal half of flank, often forming big blotch when coalesced. Dorsum between head and dorsal‐fin origin dark brown, with some depigmented small spots; venter brownish yellow, with diffuse pale brown spots. Side and dorsal surface of head dark brown, opercular and interopercular patches of odontodes pale brownish yellow. Barbels hyaline with some dark brown blotches irregularly arranged. Iris black. Fins hyaline with yellowish pigmentation concentrated on basal portion of rays and irregular transverse rows of small dark brown spots; bright white pigmentation over basal portion of pectoral and pelvic fins.

#### 3.5.8 Colouration in alcohol (Figure 1)

Similar to colouration in life, but paler.

### 3.6 Etymology

The specific name *jussariensis* refers to Jussari Municipality where the new species was discovered and collected.

### 3.7 Distribution and habitat (Figures 7 and 8)

**FIGURE 7:**
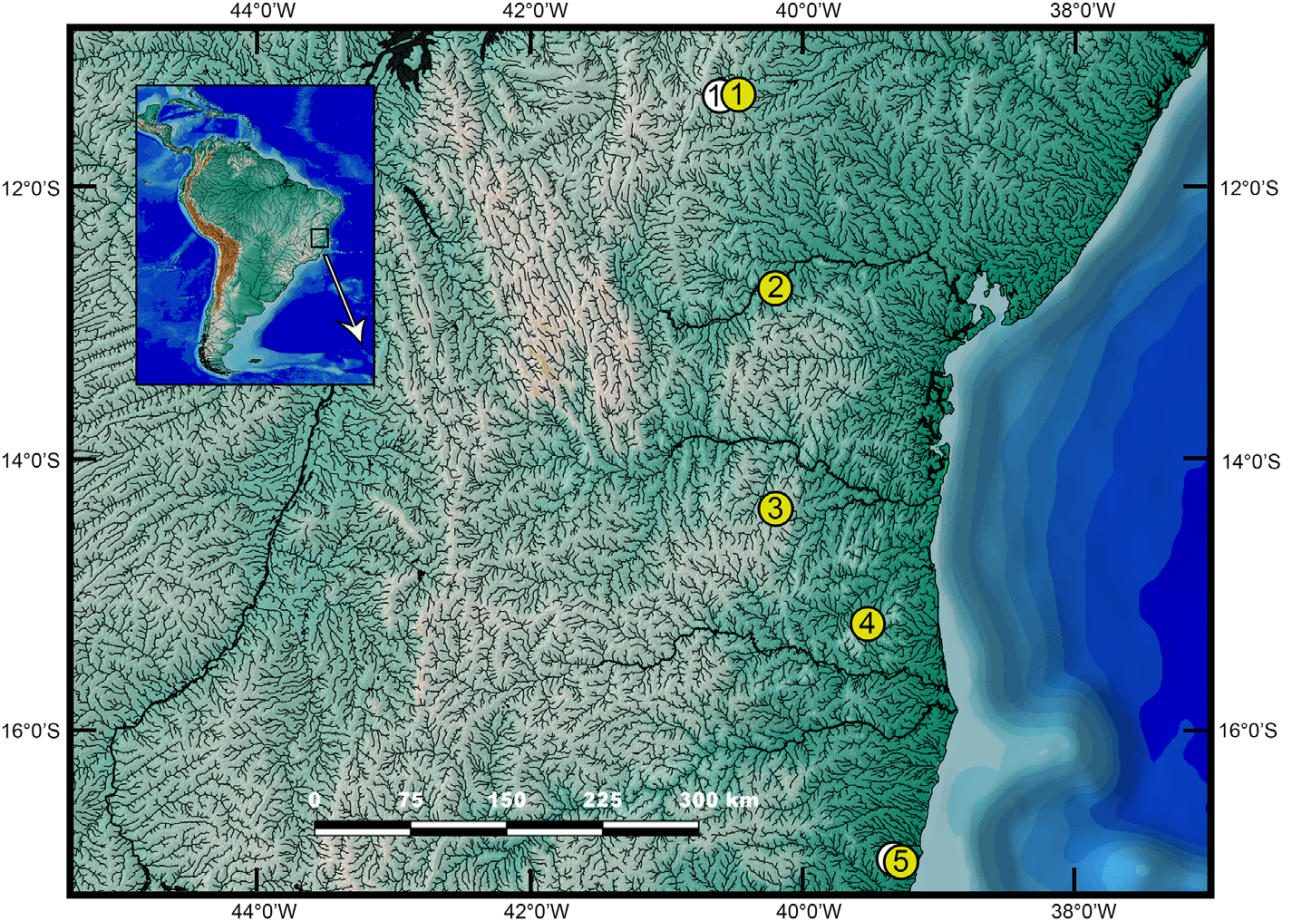
Geographical distribution of *Ituglanis* species from Northeastern Mata Atlantica ecoregion. 1, *Ituglanis payaya*; 2, *Ituglanis paraguassuensis*; 3, *Ituglanis agreste*; 4, *Ituglanis jussariensis*; 5, *Ituglanis cahyensis*. Yellow circles represent holotype localities, and white circles represent paratype localities.

**FIGURE 8:**
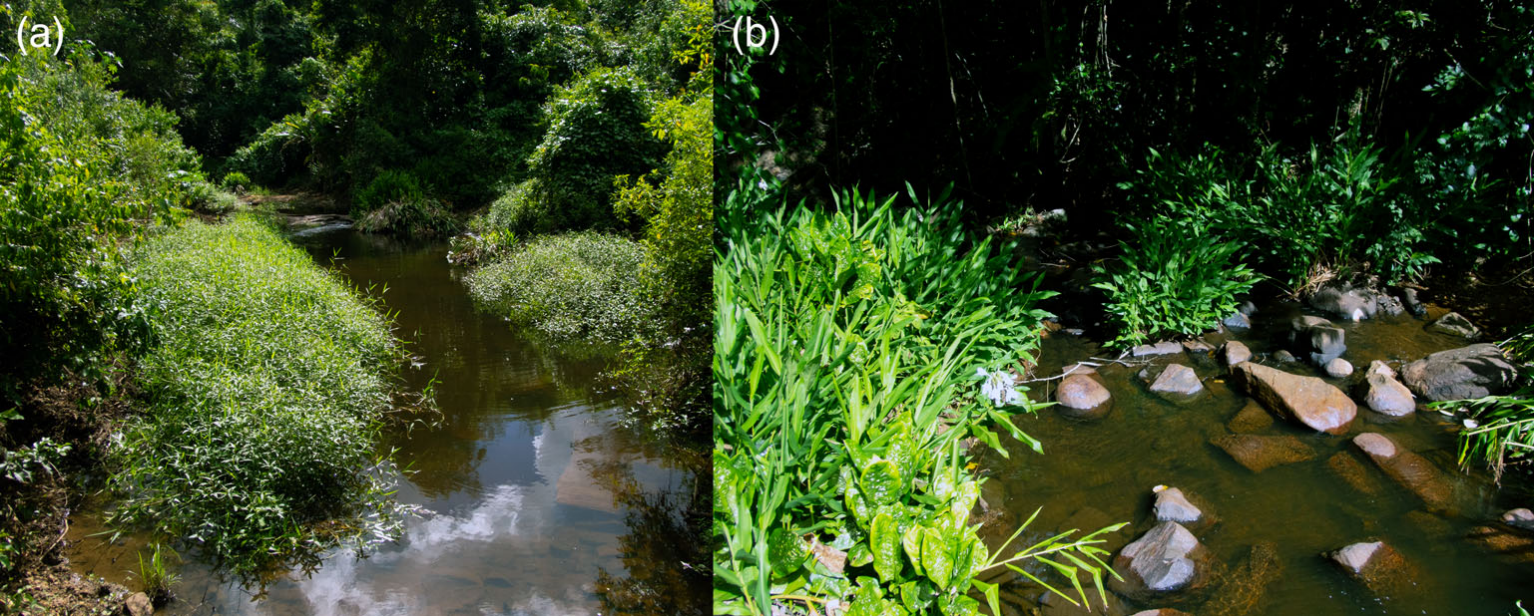
(a,b) Rio São Roque drainage, Rio Piabanha basin, Jussari Municipality, Bahia State, Brazil, the type locality of *Ituglanis jussariensis*.

*I. jussariensis* is known only from its type locality in Ribeirão São Roque, a stream tributary of the Rio Piabanha, an affluent of the Rio Colônia basin, in Jussari Municipality, Bahia State (Figure 7). The type locality is a clearwater stream, about 30–50 cm deep and 1.5–2.0 m wide. Specimens were collected associated with marginal vegetation, but mainly in the roots of the invasive species *Hedychium coronarium* J. Koenig, which was abundant along the stream banks (Figure 8). The surrounding vegetation at the collection site corresponds to the edge of a preserved forest fragment.

### 3.8 Conservation status

*I. jussariensis* has a maximum EOO estimated in 193.14 km^2^, corresponding to the surface of Ribeirão São Roque. Despite extensive sampling in several sites around the type locality, the species was found exclusively at its original collection site. This could be related to its habitat specificity, as it tends to remain hidden among the roots of aquatic plants in high oxygenated clearwater streams. Additionally, Ribeirão São Roque is surrounded by farms, and an apparent deforestation process seems to have begun around this locality. Although we adopt a conservative approach and suggest that *I. jussariensis* should be listed as data deficient (DD), as further efforts are necessary to understand its distribution better.

We assessed the conservation status of *Ituglanis* species from Bahia State using the IUCN Red List criteria. The classification proposed for each conservation status, along with the EOO for each species, is summarized in Table 2, and we discuss each in the discussion section. Additionally, we provide all the co‐ordinates used to calculate the EOO for each species, along with the relevant voucher information in Table 3.

**TABLE 2:** List of *Ituglanis* species of Bahia State and their conservation status.

*Ituglanis* species	Reference	Last assessment	New suggestion	Number of known co‐ordinates	AOO	EOO – calculated using hydrobas lv12
*Ituglanis agreste*	Lima et al. (2013), Silva et al. (2020), Vieira‐Guimarães et al. (2024), This study	IUCN 2018 DD	EN B1 ab(ii,iii)	2	8 km^2^	171.16 km^2^
*Ituglanis cahyensis*	Vieira‐Guimarães et al. (2024), Sarmento‐Soares et al. (2006), This study.	IUCN 2022 NT B1b(iii,v)	EN B1b(iii,v)	2	8 km^2^	158.69 km^2^
*Ituglanis jussariensis*	This study	‐	DD	1	4 km^2^	193.14 km^2^
*Ituglanis paraguassuensis*	Campos‐Paiva and Costa (2007), Datovo et al. (2016), Vita et al. (2020), Santos et al. (2023), de Pínna and Wosiacki (2003), Vieira‐Guimarães et al. (2024), This study	IUCN 2018 NT B1b(iii)	NT B1b(iii)	9	28 km^2^	2442.5 km^2^
*Ituglanis payaya*	Costa et al. (2021), Silva et al. (2020), Sarmento‐Soares et al. (2011), This study	IUCN 2018 DD	EN B1 ab(iii)	3	8 km^2^	448.38 km^2^

**TABLE 3:** List of vouchers and co‐ordinates of each *Ituglanis* species from Bahia State.

Species	Voucher	Co‐ordinates
*Ituglanis agreste*	MNRJ 40196*;MNRJ 40197*; UFBA 7134; UFRN 29	14°22′08.95″S 40°11′45.33″w
	MZUSP 102535	14.4S 40.2 W
*Ituglanis cahyensis*	MNRJ 28404*; MNRJ 28405*	16°57′48″S 39°16′33″W
	MNRJ 28406*	16°56′25″S 39°19′48″W
*Ituglanis jussariensis*	UFRJ 14089*; UFRJ 14135*; UFRJ 14160*; UFRJ Holotype*	15°12′53.02″S 39°31′13.63″W
*Ituglanis paraguassuensis*	MBML 2600, MBML 2599	Aprox 12°14′10.34″S 41°9′38.74″W
	MZUSP 63138	Aprox 12°22′27.43″S 41°29′45.27″W
	MZUSP 120493	12°30′17.00″S 41°12′27.00″W
	MZUSP 120510	12°21′23.28″S 41°32′25.70″W
	MNRJ 22765*; MNRJ 22763*	12°14′43.00″S 41°9′48.00″W
	MNRJ 22757*	Aprox 12°14′10.00″S 41°9′44.00″W
	LBP 30711*	12°22′19.00″S 41°31′6.30″W
	UFRJ 11153*; UFRJ 10709*	12°06′44″S 41°07′14″W
	LIRP 5834*; UFRJ 7209*; UFRJ 7282*; USNM 301016*; LIRP 5780*; UFRJ 7209*	12°45’S 40°12’W
*Ituglanis payaya*	MNRJ 36665*; ANSP 190965; MBML 2560; MNRJ 36666*; MZUSP 88164;	11°20′19.5″S 40°36′21.9″W
	UFBA 5286; UFBA 5284;	11°19′41.1″S 40°28′11.2″W
	UFRJ 12270*; UFRJ 12590*;	11°19′43″S 40°28′13″W

*Note*: Each voucher marked with * was analysed by the authors in person.

## 4 DISCUSSION

*I. jussariensis* inhabits Ribeirão São Roque, a small stream part of Rio Colônia basin, an isolated coastal drainage in southern Bahia State, a region that is part of the Northeastern Mata Atlantica freshwater ecoregion (NMA). This ecoregion, proposed by Abel et al. (2008), includes three distinct biomes: the Atlantic Forest, where the new species was discovered, Cerrado and Caatinga. Among these, the Atlantic Forest is significantly more humid when compared to the other two biomes. The five species of *Ituglanis* recorded for Bahia State occur in different zones of this ecoregion. Although *I. payaya* and *I. paraguassuensis* are found within the Caatinga region of the Chapada Diamantina, *I. agrestes* is distributed in a transition zone between remnants of the Atlantic Forest and the Caatinga, whereas *I. cahyensis* occurs in remnants of the Atlantic Forest.

Preliminary molecular analyses for ongoing studies indicate that *I. jussariensis* may form a clade with *I. paraguassuensis* and *I. payaya*. This potential relationship, linking species from distinct biomes, would be particularly relevant given similar patterns observed in other fish groups from the same ecoregion (Camelier & Zanata, 2014). However, it is not possible to perform a more comprehensive discussion of the relationships among *Ituglanis* species from Bahia, as molecular data for *I. agrestes* and *I. cahyensis* are still lacking.

**FIGURE 9:**
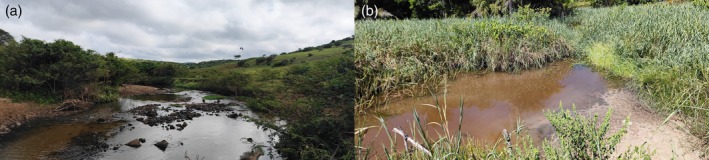
Type locality of (a) *Ituglanis agrestes* and (b) *Ituglanis cahyensis*. Photographs taken during a field survey in February 2024.

Given the significant environmental threats affecting the habitats of these species and the limited data available, reassessing the conservation status of each *Ituglanis* species from Bahia State is essential. The conservation status of *I. agrestes*, *I. cahyensis*, *I. paraguassuensis* and *I. payaya* was proposed in different years (2018 and 2022), and it is presented in Table 2. In this study, we propose a status for *I. jussariensis* and an updated status for the other *Ituglanis* species from Bahia based on the most recent data available. The conservation status of *I. agrestes* was assessed in 2018 by the IUCN Red List of Threatened Species and classified as DD. Herein, we calculated an EOO of 171.16 km^2^ and recommend updating the status to endangered (EN) based on the criteria B1ab (ii,iii). *I. agrestes* is known to occur in only two localities (to fit the sub criteria ‘a’, need to be above 5); its EOO calculation is above 500 km^2^; and it has a notably lower quality of its habitat conservation. Its type locality, a tributary of the Rio Gongagi drainage, has signs of sewage dumping and human alteration (several small and rudimentary dawns were constructed to canalize water). No species that were reported for that locality (Lima et al., 2013) were found in a field survey conducted in March 2024 (Figure 9a).

*I. cahyensis* has its conservation status last evaluated in 2022 by the IUCN Red List of Threatened Species and classified as near threatened (NT) under the criteria B1b(iii,v). We suggest an EN status, as it continues to meet the requirements of the criteria b(iii, v), but its habitat also appears to be suffering from a decline in water quality, as no fish species have been found in the region, and the environment around the river is under a deforestation process. Additionally, this species is known from only two localities, also fitting the sub‐criteria a. Previous conservation status evaluations from Secretaria do Meio Ambiente do Estado da Bahia (SEMA, 2017) and national assessment (MMA, 2014) also classified the species as EN. Considering the absence of recent records and the progression of the environmental degradation observed in our last survey to its type locality in March 2024, we recommend classifying it as EN (Figure 9b).

*I. paraguassuensis* had its conservation status evaluated in 2018 by the IUCN Red List of Threatened Species and was classified as NT [B1 b(iii)]. We agree with that classification and propose to maintain that status. *I. payaya* was also assessed in 2018 and classified as DD. Herein, we suggest a modification to EN status, as it meets the criteria B1ab(iii) as it occurs in the Cerrado biome, a region undergoing intense deforestation that could lead to the loss of their habitat.

Finally, the conservation status of *I. jussariensis* is being assessed for the first time. We recommend classifying this species as DD. Although a deforestation process surrounds its only known locality, and its actual EOO is less than 500 km^2^, these factors alone would suggest the species could be at least classified as EN. However, due to the lack of comprehensive data regarding its distribution and potential threats, further efforts are needed to better assess its conservation status. It is important to note that Cetra et al. (2010) shed light on the possible restricted distribution of *I. jussariensis* (*Ituglanis* sp. in their paper) and suggested an expansion of REVIS Serra do Baixão, a conservation unit near to its type locality, to ensure the protection of this species and others caught in nearby areas.

In conclusion, our study highlights the urgent need for targeted conservation efforts in the Northeastern Mata Atlantica freshwater ecoregion, particularly considering the ongoing habitat degradation and the limited distribution of the evaluated species. More field surveys and molecular data acquisition are key steps to accurately delineate conservation priorities for the genus *Ituglanis* in Bahia.

## AUTHOR CONTRIBUTIONS

Paulo J. Vilardo, Axel M. Katz and Wilson J.E.M. Costa conceptualized the study and generated the data. Paulo J. Vilardo analysed the data and prepared drafts of the manuscript. Axel M. Katz and Wilson J.E.M. Costa contributed to substantial revisions of the manuscript.

## FUNDING INFORMATION

This study was supported by CNPq (Conselho Nacional de Desenvolvimento Científico e Tecnológico – Ministério de Ciência e Tecnologia – grant number 140689/2022‐2 to Paulo J. Vilardo; 304755/2020‐6 to Wilson J.E.M. Costa) and Fundação Carlos Chagas Filho de Amparo à Pesquisa do Estado do Rio de Janeiro [FAPERJ; grant E‐26/201.213/2021 to Wilson J.E.M. Costa, E‐26/202.005/2020 to Axel M. Katz; E‐26/203.644/2024 (303752) to Paulo J. Vilardo]. This study was also supported by CAPES (Coordenação de Aperfeiçoamento de Pessoal de Nível Superior – Finance Code 001) through the Programa de Pós‐Graduação em Genética/UFRJ.

## Supporting information


**Supporting information S1.** List of materials examined for the analysis of morphological characters. C&S means cleared and stained specimens, SL means standard length and NM means not measured. List follows the pattern: species name authors, year. Country, state, municipality or locality described in collection record: voucher, number of samples, measures. CAS, California Academy of Sciences (San Francisco, USA); CICCAA, Centre of Agrarian and Environmental Sciences, Federal University of Maranhão (Maranhão, Brazil); CICM, Coleção Ictiológica Morevy Cheffe, Grupo Especial de Estudo e Proteção do Ambiente Aquático do Rio Grande do Sul (Rio Grande do Sul, Brazil); FMNH, Field Museum of Natural History (Chicago, USA); LBP, Laboratório de Biologia de Peixes, UNESP‐Botucatu (São Paulo, Brazil); NMW, Naturhistorisches Museum, (Wien, Austria); MNRJ, Museu Nacional do Rio de Janeiro (Rio de Janeiro, Brazil); UFRJ, Institute of Biology, Federal University of Rio de Janeiro (Rio de Janeiro, Brazil).
