# Supplementary material for: New insights into Ituglanis (Siluriformes: Trichomycteridae) diversity in Bahia State, eastern Brazil: Description of a new species and conservation status reappraisal
Source: J Fish Biol. 2025 Oct 5;108(1):387–97. doi: 10.1111/jfb.70246 (PMC13033970; doi:10.1111/jfb.70246)
Supplement: Supplementary file 1 — Supporting information S1. List of materials examined for the analysis of morphological characters. C&S means cleared and stained specimens, SL means standard length and NM means not measured. List follows the pattern: species name authors, year. Country, state, municipality or locality described in collection record: voucher, number of samples, measures. CAS, California Academy of Sciences (San Francisco, USA); CICCAA, Centre of Agrarian and Environmental Sciences, Federal University of Maranhão (Maranhão, Brazil); CICM, Coleção Ictiológica Morevy Cheffe, Grupo Especial de Estudo e Proteção do Ambiente Aquático do Rio Grande do Sul (Rio Grande do Sul, Brazil); FMNH, Field Museum of Natural History (Chicago, USA); LBP, Laboratório de Biologia de Peixes, UNESP‐Botucatu (São Paulo, Brazil); NMW, Naturhistorisches Museum, (Wien, Austria); MNRJ, Museu Nacional do Rio de Janeiro (Rio de Janeiro, Brazil); UFRJ, Institute of Biology, Federal University of Rio de Janeiro (Rio de Janeiro, Brazil). [file JFB-108-387-s001.docx]

***Ituglanis agrestes* Lima, Neves & Campos-Paiva, 2003** Brazil, Bahia State, Rio de Contas basin: MNRJ 40196, holotype, 40.5mm SL; MNRJ 40197, 5 paratypes, 32.4–40.8 mm SL. ***Ituglanis amazonicus* (Steindachner 1882)**: Brazil, Acre State, Rio Branco Municipality: LBP 201, 1, 51.42 mm SL. Brazil, Amazonas State, Amazonas River basin: LBP 18002, 1 (DNA), NM. Brazil, Amazonas State, Codajás Municipality: NMW 43306, holotype (X-RAY), 49.7 mm SL. Brazil, Amazonas State, Madeira/Amazonas River basin: LBP12012, 2, 30.0–34.4 mm SL; LBP 12104, 6, 39.9–42.7 mm SL. Brazil, Amazonas State, Rio Purus basin: UFRJ 13662, 2 (C&S), NM; UFRJ 5866, 10, 25.4–38.3 mm SL. Brazil, Amazonas State, Rio Preto da Eva river drainage: UFRJ 10427, 1 (DNA), NM. Brazil, Maranhão State, Itapecuru River basin: CICCAA 02888, 3, NM. Brazil,Maranhão State, Rio Tocantins basin: CICCAA 01851, 1, NM. Brazil, Mato Grosso State, Araguaia River basin: LBP1511, 1, 50.5 mm SL. Brazil, Pará State, Guamá River Basin: LBP 9298, 2, 41.1–43.3 mm SL; UFRJ 4955, 1, NM. Brazil, Pará State, Tapajós/Amazonas River basin: LBP 16251, 1, 48.2 mm SL. Brazil, Pará State, Xingu/Amazonas River basin: LBP 16633, 3, 38.1–47.1mm SL. Brazil, Pará State, Rio Tocantins/Amazonas River basin: LBP 17650, 6, 31.3–40.2mm SL; LBP 17651, 1, 47.6 mm SL. Brazil, Pará State, Teles Pires/Amazonas River basin: LBP 24826, 20, 30.1–49.6 mm SL; LBP 33337, 1, 96.47 mm SL. Brazil, Rondônia State, Madeira/Amazonas River basin: LBP10870, 4, 36.22–62.56 mm SL; LBP10967, 2, 36.1–48.6 mm SL; LBP 11003, 3, 39.9–68.9 mm SL; LBP 11036,1, 35.9mm SL. ***Ituglanis amphipoptamus* Mendonça, Oyakawa & Wosiacki, 2018:** All from Brazil. São Paulo State, Sete Barras Municipality: UFRJ 12548, 1, 41.4 mm SL; UFRJ 12688, 2(C&S), NM. São Paulo State, Jacupiranga Municipality: UFRJ 8730, 1, 63.0 mm SL. São Paulo State, Itapeúna Municipality: LBP 7416, 7, 41.2–55.2 mm SL. ***Ituglanis australis* Datovo & de Pinna, 2014** All from Brazil. Rio Grande do Sul State, Aceguá Municipality: UFRJ 14260, 3, 33.0–34.0 mm SL. Rio Grande do Sul State, Rio Piratini basin: CIMC 1442, 5, 39.5–59.9 mm SL; CIMC 1519, 8, 37.7–64.8 mm SL; CIMC 1542, 2, 54.0–66.7 mm SL; CIMC 1982, 3, 47.2–68.4 mm SL; CIMC 2601, 1, 68.2 mm SL; UFRJ 13593, 2(C&S), NM. Rio Grande do Sul State, Rio Jaguarão basin: CIMC 8022, 2, 43.3–52.2mm SL. Rio Grande do Sul State, Rio Camaquã basin: CIMC 1712, 1, 53.8 mm SL. Rio Grande do Sul State, Herval Municipality: UFRJ 13374, 2, NM; UFRJ 13428, 1, NM. ***Ituglanis* *boitata* Ferrer, Donin & Malabarba 2015:** All from Brazil. Rio Grande do Sul State, Agudo Municipality: LBP 14546, 2, 44.9–49.9 mm SL. Rio Grande do Sul State, Maquiné Municipality: UFRJ 10583, 1(C&S), 60.7 mm SL; UFRJ 13324, 4, NM; UFRJ 13339, 3, 103.5–112.8 mm SL; UFRJ 13750, 1 (C&S), NM. Santa Catarina State, Ararangua River Basin: UFRJ 13340, 1, 99.7 mm SL; UFRJ 12197, 2, 39.3–53.6 mm SL. Santa Catarina State, Jacinto Machado Municipality: UFRJ 13448, 5, 34.4–39.7 mm SL; UFRJ 12881, 2, 106.8–149.6 mm SL. Santa Catarina State, Orleans Municipality: UFRJ 13341, 1, 103.1 mm SL; UFRJ 13433, 1, 35.0 mm SL; UFRJ 13451, 6, 33.9–60.9 mm SL; UFRJ 13453, 4, 32.7–56.7 mm SL; UFRJ 13468, 1(C&S), NM. Santa Catarina State, Praia Grande Municipality: UFRJ 13450, 2, 46.2–48.8 mm SL; UFRJ 13452, 2, 66.0–85.7 mm SL, UFRJ 13687, 2, NM. Santa Catarina State, Siderópolis Municipality: UFRJ 10673, 1, NM; UFRJ 13323, 4, NM; UFRJ 14340, 1, NM, UFRJ 13455, 1, 38.0 mm SL. Santa Catarina State, Treviso Municipality: UFRJ 12191, 2, NM; UFRJ 13449, 3, 27.1–50.2 mm SL. ***Ituglanis cahyensis* Sarmento-Soares, Martins-Pineiro, Arranda & Chamon, 2006**. All from Brazil. Bahia State, Prado municipality: MNRJ 28404, 1 paratype, 48.6 mm SL; MNRJ 28405, 2 paratypes (C&S), 41.3–48.6 mm SL; MNRJ 28406, 1 paratype, 28.6mm SL. ***Ituglanis compactus*** **Castro & Wosiacki, 2017.** All from Brazil. Amapá State, Laranjal do Jari Municipality: UFRJ 8859, 4, NM. ***Ituglanis* *eichorniarum* (Miranda-Ribeiro, 1912).** Brazil, Mato Grosso State, Cáceres Municipality: MNRJ 780, 1 lectotype, 31.3 mm SL. Brazil, Mato Grosso State, Itiquira Municipality: LBP 1916, 5, 36.4–41.1mm SL. Brazil, Mato Grosso State, Porto Cercado Municipality: UFRJ 5474, 1, NM; UFRJ 5608, 1 (c&s), 24.3 mm SL. Brazil, Mato Grosso State, Tangará da Serra Municipality: LBP 4686, 1, 36.29 mm SL. ***Ituglanis goya* Datovo, Aquino & Langeani, 2016.** All from Brazil. Goiás State, Cavalcante Municipality: MNRJ 11486, 35, 38.2–56.8 mm SL; LBP 17131, 1, 59.8mm SL; LBP 19296, 10, 32.9–52.1mm SL; LBP 19297, 1, 51.2 mm SL; LBP 19299, 1, 57.8 mm SL; LBP 19471, 1, 46.8 mm SL; LBP 19472, 1, 56.6 mm SL. Goiás State, Colinas do Sul Municipality: MNRJ 11485, 50, 38.4–57.9 mm SL. Goiás State, Mimoso Municipality: UFRJ 9651, 2, 50.7–53.3 mm SL. Goiás State, Minaçu Municipality: MNRJ 11487, 2, 52.4–55.0 mm SL; MNRJ 11488, 5, 53.1–67.2 mm SL; MNRJ 11489, 13, 55.2–65.1 mm SL; MNRJ 11490, 1, 75.2 mm SL; MZUSP 53222, 8, 51.0–53.7 mm SL; UFRJ 13665, 2, NM. Goiás State, Monte Alegre Municipality: MZUSP 40654, 19, 30.5–46.9 mm SL. Goiás State, Nova Roma Municipality: LBP 19273, 2, 44.9–51.9 mm SL. Goiás State, São Gabriel de Goiás Municipality: UFRJ 12687, 2 (C&S), NM; UFRJ 8330, 3, 35.9–53.75 mm SL. Goiás State, Teresina de Goiás Municipality: LBP 19315, 1, 35.3 mm SL. ***Ituglanis herberti* (Miranda-Ribeiro, 1940):** Brazil, Mato Grosso State, Rio Bodoquena: MNRJ 1429, 5, paralectotypes, 52.5–64.2 mm SL. ***Ituglanis ina* Wosicaki, Dutra & Mendonça, 2012:** Brazi, Pará State, Serra dos Carajás: UFRJ 13971, 1, 55.23mm SL; UFRJ 13972, 2 (C&S), NM. ***Ituglanis macunaima* Datovo & Landim, 2005:** All from Brazil. Goiás State, Aruanã Municipality: UFRJ 1587, 1, 34.0 mm SL; UFRJ 1676, 16, 18.9–38.1 mm SL; UFRJ 12689, 2 (C&S), NM. Goiás State, Novo Planalto Municipality: UFRJ 1468, 1, 30.9 mm SL. Mato Grosso State, Vale dos Sonhos Municipality: LBP 5745, 1, NM. ***Ituglanis* *paraguassuensis* Campos-Paiva & Costa 2007:** All from Brazil. Bahia State, Utinga Municipality: UFRJ 7209, 1 paratype, 36.5 mm SL; UFRJ 7282, 1 paratype, 39.6 mm SL (c&s); UFRJ 10709, 15, 25.7–43.6 mm SL. Bahia State, rio Paraguaçu basin: USNM 301016, 5 (2 c&s) paratypes, 30.3–41.6 mm SL. Bahia State, Iraquara Municipality: LBP 30711, 4, 30.3–36.8mm SL; LBP 34055, 1, 29.5mm SL. ***Ituglanis* *parahybae* (Eigenmann 1918):** All from Brazil. Rio de Janeiro State, Aldeia Velha Municipality: UFRJ 0761, 1, 42.2 mm SL; UFRJ 0704, 1, 38.6 mm SL; UFRJ 3358, 3, NM; UFRJ 7204, 2, 27.8–34.9 mm SL; UFRJ 1103, 1(c&s), NM. Rio de Janeiro State, Conceição de Macabu Municipality: LBP 10730, 1, 42.1mm SL; Rio de Janeiro State, São João da Barra Municipality, Rio Paraíba do Sul basin: CAS 58576, holotype (X-RAY), 27.9 mm SL. ***Ituglanis payaya* Sarmento-Soares, Zanata & Martins-Pinheiro, 2011***:* Brazil, Bahia State, Jacobina Municipality: UFRJ 12590, 6(C&S), NM; UFRJ 12554, 20, 15.2–51.1 mm SL. ***Ituglanis parkoi* (Miranda-Ribeiro, 1944):** Amazonas State, Benjamin Constant Municipality: MNRJ 3849, holotype, 142.20 mm SL. ***Ituglanis* *proops* (Miranda-Ribeiro 1908):** Brazil: Paraná State, Cerro Azul Municipality: UFRJ 13460, 1 (C&S), NM; UFRJ 13462, 1(C&S), NM. São Paulo State: USNM 38785, 1, 55.3 mm SL. UFRJ 14024, 2, 32.5–47.0 mm SL. ***Ituglanis ramiroi* Bichuette & Trajano, 2004**: All from Brazil. Goiás State, São Domingos Municipality: LBP 15293, 3, NM.
